# Medicarpin, a legume phytoalexin sensitizes myeloid leukemia cells to TRAIL-induced apoptosis through the induction of DR5 and activation of the ROS-JNK-CHOP pathway

**DOI:** 10.1038/cddis.2014.429

**Published:** 2014-10-16

**Authors:** R Trivedi, R Maurya, D P Mishra

**Affiliations:** 1 Cell Death Research Laboratory, Division of Endocrinology, CSIR-Central Drug Research Institute, Lucknow 226031, India; 2 Medicinal Process Chemistry Division, CSIR-Central Drug Research Institute, Lucknow 226031, India

## Abstract

Tumor necrosis factor *α*-related apoptosis-inducing ligand (TRAIL) is a promising anticancer agent with cancer cell-selective cell death inducing effect. However, the major limitation in the usage of TRAIL as a chemotherapeutic agent is the development of TRAIL resistance in many cancer types including myeloid leukemia. In this study, we report for the first time that Medicarpin (Med), a naturally occurring phytoalexin sensitizes myeloid leukemia cells to TRAIL-induced apoptosis. Combination of Med and TRAIL induced significantly higher apoptosis compared with that of the individual treatments of either agent alone through activation of both the extrinsic and the intrinsic cell death pathways characterized by the activation of caspases 8, 9, 3, and 7. Med treatment downregulated antiapoptotic proteins (Survivin, Bcl2, Bcl-xL, XIAP, and c-FLIP), upregulated pro-apoptotic proteins (Bax, Cytochrome C, Smac/Diablo, Bid, truncated Bid (tBid), p-eIF2*α*, Bip, and CHOP (CCAAT-enhancer binding protein homologous protein)), induced G2/M cell-cycle arrest, and increased the expression of the functional TRAIL receptor DR5 through activation of the ROS-JNK-CHOP pathway. Gain and loss of function studies clearly indicated that DR5 expression was critical for Med-induced TRAIL sensitization. The Med-induced TRAIL sensitization did not involve the NFkB signaling pathway or redistribution of DR5 in lipid rafts. The concomitant treatment with Med and TRAIL showed robust apoptotic effects in primary myeloid leukemia cells but had no toxic effects in primary human peripheral blood mononuclear cells (PBMCs). In conclusion, our results suggest that Med sensitizes myeloid leukemia cells to TRAIL-induced apoptosis through the upregulation of DR5 through activation of the ROS-JNK-CHOP pathway.

Myeloid leukemia is a hematologic malignancy characterized by aberrant hematopoiesis with rapid proliferation of the undifferentiated and immature blood cells of the myeloid lineage.^1^ The conventional therapeutic strategies for myeloid leukemia are limited by systemic side effects, development of chemoresistance, and poor survival outcomes.^2^ Therefore, it is necessary to look for novel therapeutic agents and strategies for this dreadful disease.

Therapy failure and chemo-resistance in myeloid leukemia cells are attributed to several mechanisms including resistance to the death receptor (DR)-mediated apoptosis.^3^ The tumor necrosis factor-related apoptosis-inducing ligand (TRAIL) is a promising anticancer agent characterized by its cancer cell-specific pro-apoptotic action mediated through the DRs.^4^ However, its therapeutic potential is severely compromised by acquired TRAIL resistance through the downregulation of DRs and adaptor proteins or the increased expression of antiapoptotic proteins in myeloid leukemia cells.^5^ Therefore, it is important to identify novel agents that can sensitize these TRAIL-resistant cells and could be combined therapeutically with TRAIL to amplify its pro-apoptotic effects.^5^

In search of novel yet non-toxic TRAIL sensitizing agents, numerous studies have focused their attention on natural agents that could potentiate TRAIL-mediated apoptotic effects at physiologically attainable concentrations.^6^ The plant phytoalexins are a class of low molecular weight compounds with potent anticancer activities exerted through inhibition of cancer cell proliferation, invasion, and metastasis.^7^ Medicarpin (Med), a legume phytoalexin has excellent oral bioavailability and potent antiproliferative activity against breast cancer and acute myeloid leukemia (AML) cells.^8,9^ Med also inhibits the oncogenic NFkB signaling by attenuating the TNF-*α*-induced nuclear translocation of p65.^8^

In this study, we hypothesized that Med does not only inhibit cancer cell proliferation but also sensitizes cells to undergo DR-mediated apoptosis and explored whether it would sensitize the myeloid leukemia cell lines to TRAIL-induced apoptosis. We further attempted to elucidate the molecular mechanism associated with this effect.

## Results

### Med sensitizes myeloid leukemia cells to TRAIL-induced cell death

**Figure 1:**
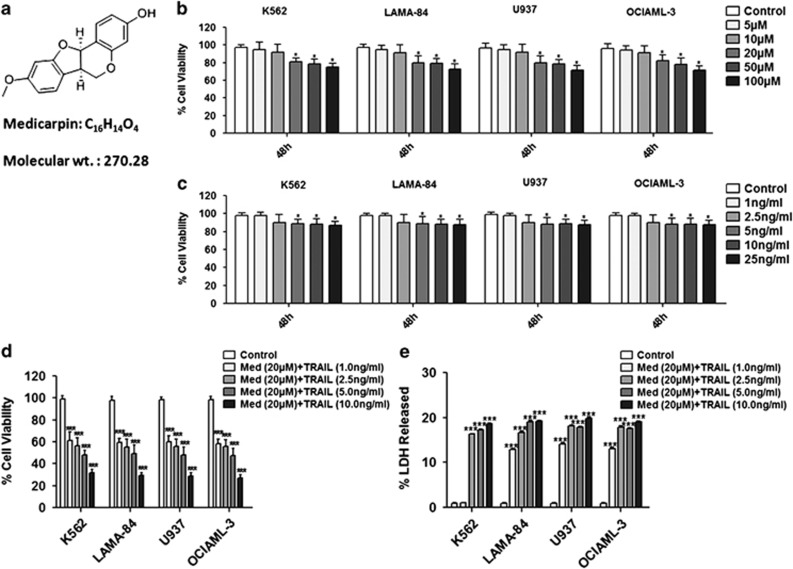
Med potentiates TRAIL-induced apoptosis in leukemia cells. (**a**) Chemical structure of Med. (**b**) K562, LAMA-84, U937, and OCIAML-3 cells were treated with indicated doses of Med for 48 h. Cell viability was then analyzed by cytotoxicity assay using CCK-8 as described under ‘Materials and Methods'. (**c**) K562, LAMA-84, U937, and OCIAML-3 cells were treated with indicated doses of TRAIL for indicated time points and cell viability was then analyzed by cytotoxicity assay using CCK-8 as described under ‘Materials and Methods'. (**d**) K562, LAMA-84, U937, and OCIAML-3 cells were treated with 20 *μ*M Med and indicated doses of TRAIL for 48 h. Cell viability was then analyzed by cytotoxicity assay using CCK-8 as described under ‘Materials and Methods'. (**e**) K562, LAMA-84, U937 and OCIAML-3 cells were treated with 20 *μ*M Med and indicated doses of TRAIL for 48 h. Released LDH was measured as described under ‘Materials and Methods'. Data are presented as mean±S.D. of three independent experiments. *P>0.05, **P>0.001, ***P>0.0001 in treated groups *versus* control group

In the first set of experiments to assess the TRAIL sensitizing effect of Med (Figure 1a), myeloid leukemia cells were incubated in culture media supplemented with dimethyl sulfoxide (DMSO) or various concentrations of Med (Figure 1b) or TRAIL (Figure 1c) for 48 h. The cell viability was quantified using the CCK-8 assay. Both Med and TRAIL induced a slight dose-dependent decrease in cell viability in myeloid leukemia cells (Figures 1b and c; Supplementary Figure S1a and b). Med alone significantly reduced cell viability at higher doses and showed sensitization with various doses of TRAIL in all the cell lines at a dose of 20 *μ*M. Concomitant treatment of TRAIL and Med resulted in a significant reduction in cell viability in all the four tested cell lines (Figure 1d). In the next set of experiments, myeloid leukemia cells were preincubated with DMSO or Med alone for 24 h and subsequently treated with TRAIL for an additional 24 h. However, the sequential treatment of Med and TRAIL had similar effects on cell viabilities (data not shown) to the concomitant treatment of these agents. On the basis of these experiments, sensitization to TRAIL-induced apoptosis was observed in all the tested myeloid leukemia cell lines. Therefore, we chose the doses of 20 *μ*M of Med with 2.5 ng/ml of TRAIL for further mechanistic experiments.

As TRAIL is supposed to mainly act through induction of cell death, therefore we studied the lactate dehydrogenase (LDH) released into the culture medium to assess cell death induction in our experimental setting. Consistent with the observed antiproliferative effect, treatment with the combination of Med and TRAIL induced a marked LDH release in the treated cells (Figure 1e; Supplementary Figure S1c and d). These results collectively indicated that Med sensitizes myeloid leukemia cells to TRAIL-induced cell death.

### TRAIL-induced apoptosis in Med-treated cells involves both the DR and the mitochondrial apoptotic pathways

**Figure 2:**
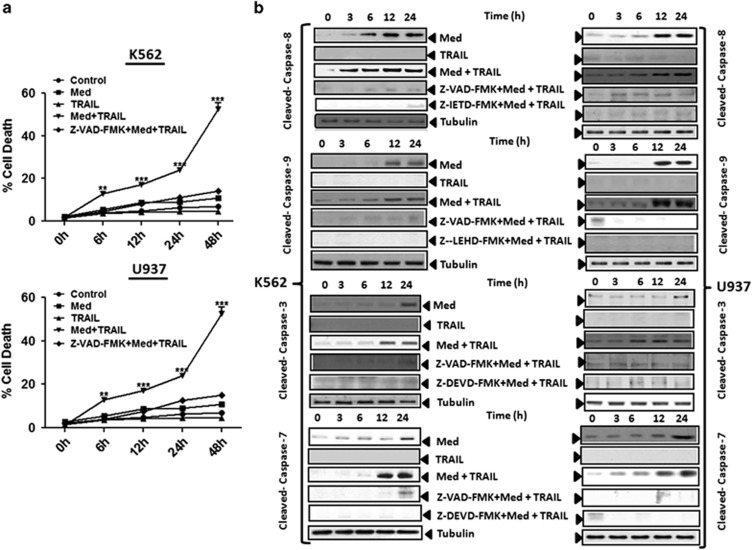
TRAIL-induced apoptosis in Med-treated cells involves both the death receptor and the mitochondrial apoptotic pathways. (**a**) K562 and U937 cells were either treated with 20 *μ*M Med or 2.5 ng/ml TRAIL or Med+TRAIL for the indicated time periods. Cells were then stained with Annexin V/7AAD and then analyzed by flow cytometry. Data are presented as mean±S.D. of three independent experiments. *P>0.05, **P>0.001, ***P>0.0001 in treated groups *versus* control group. (**b**) K562 and U937 cells were either treated with 20 *μ*M Med or 2.5 ng/ml TRAIL or Med+TRAIL or Med+TRAIL with 2 h preincubation of 50 *μ*M of pan caspase inhibitor ( Z-VAD-FMK) or specific caspase inhibitors (caspase-8:Z-IETD-FMK, caspase-9: Z-LEHD-FMK, caspase-3: Z-DEVD-FMK and caspase-7: Z-DEVD-FMK) for the indicted time periods and caspase activation was determined using western blotting analysis. Representative western blots images of three independent experiments are presented

We next assessed the effect of Med+TRAIL combination induced apoptosis in myeloid leukemia cells by measuring the sub-G0 cell population by flow cytometry. Med alone induced a statistically significant increase in the sub-G0 events in all the tested cell lines. The sub-G0 events accounted for 44.5, 43.9, 45.2, and 43.9% in K562, LAMA-84, U937, and OCIAML-3 cell populations, respectively (Supplementary Figure S1e). We further confirmed these results in K562 and U937 cells using the 7AAD/Annexin-based cell death assays. Treatments with either Med or TRAIL alone did not induce significant apoptosis in these cells, consistent with the earlier reports that myeloid leukemia cells being resistant to TRAIL-induced apoptosis.^10,11^ However, the concomitant treatment of Med and TRAIL to cells induced massive apoptosis in a time-dependent manner (Figure 2a, *Upper and Lower Panel*, Supplementary Figure S2).

We next measured caspase-3/7 (the principal effector caspases committing cells to apoptosis), caspase-8 (mediator of the DR apoptosis pathway), and caspase-9 (mediator of the mitochondrial apoptosis pathway) activities to confirm TRAIL-induced apoptosis in Med-treated cells. Med alone induced significant caspase-3/7 activity in all cell types while TRAIL in agreement with the flow-cytometry data induced minimal caspase activation. However, the combined treatment of Med and TRAIL induced a dramatic increase in the caspase-3/7 activity (Supplementary Figure S1f) in all the cell lines (7.9-fold in K5622, 7.8-fold in LAMA-84, 8.1-fold in U937, and 7.5-fold in OCIAML-3). Similarly, Med and TRAIL alone induced a modest increase in the caspase-8 and caspase-9 activity in the treated cells. However, the concomitant treatment with Med and TRAIL induced a marked increase in caspase-8 activity (6.7-fold in K562, 6.5-fold in LAMA-84, 6.4-fold in U937, and 6.2-fold in OCIAML-3 cells, Supplementary Figure S1g) and an even more pronounced increase in the caspase-9 activity (8.6-fold in K562, LAMA-84, U937, and OCIAML-3 cells, Supplementary Figure S1h). We further confirmed these results through assessment of the caspase activation through immunoblotting guided detection of cleaved caspases in K562 and U937 cells. In agreement with the earlier findings we observed that the Med+ TRAIL induced sequential activation of caspases 8, 9, 3, and 7 (Figure 2b). These results collectively indicated that both DR and mitochondrial pathways contribute to the TRAIL-induced apoptosis in Med-treated cells.

### Med downregulates the cell survival proteins and upregulates cell death associated protein expression

**Figure 3:**
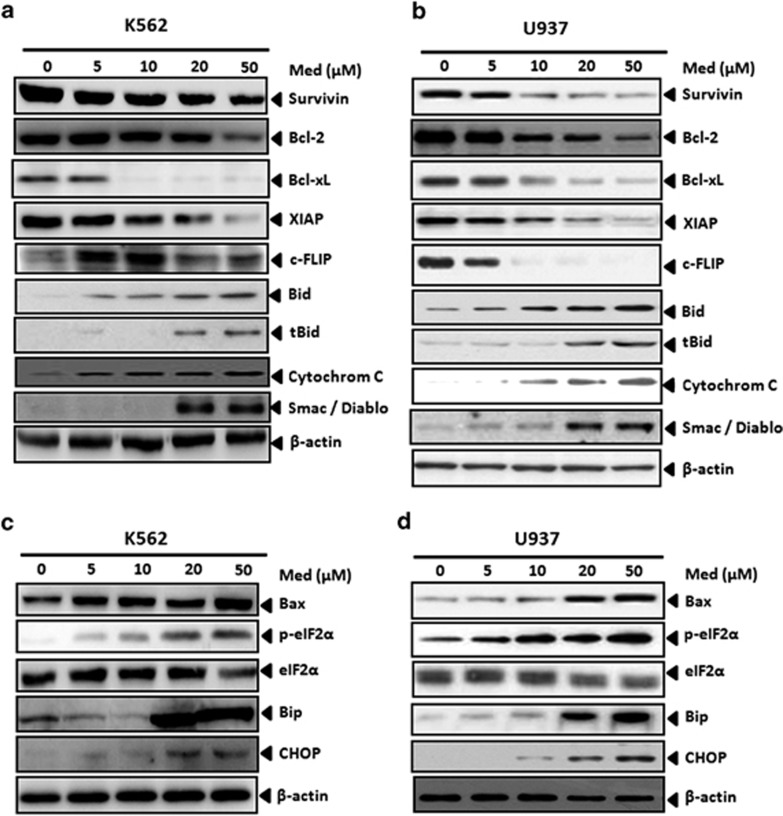
Med treatment alters pro- and antiapoptotic protein expression. K562 and U937 cells were treated with indicated doses of Med for 48 h. After treatment, whole-cell extracts were prepared and antiapoptotic proteins in both K562 (**a**) and U937 (**b**) and proapoptotic proteins in both K562 (**c**) and U937 (**d**) were analyzed by western blotting. Representative images of three independent experiments are presented

The antiapoptotic proteins such as survivin,^12,13^ Bcl-2, XIAP,^5^ and c-FLIP^14^ have been shown to regulate TRAIL-induced apoptosis. Therefore, we investigated whether Med potentiates TRAIL-induced apoptosis by regulating these cell survival proteins. K562 and U937 cells were exposed to different concentrations of Med for 48 h and then examined for the expression of these antiapoptotic proteins. Med inhibited Bcl-2, Bcl-xL, survivin, XIAP, and c-FLIP expression in both K562 and U937 cells (Figures 3a and b). We next examined whether Med could modulate the expression of pro-apoptotic proteins. We found that Med dramatically upregulated the expression of Bax, Bid, truncated Bid (tBid), cytosolic cytochrome C, and Smac/Diablo in a dose-dependent manner in both K562 (Figures 3a and c) and U937 (Figures 3b and d) cells. Med treatment induced G2/M cell-cycle arrest (Supplementary Figure S3a); however, over expression of antiapoptotic proteins (Bcl-2, BclxL) and genomic inhibition of the proapoptotic (Bax, Bid) BCl-2 family proteins completely reversed Med-induced TRAIL sensitization (Supplementary Figure S3b) indicating the critical role of mitochondrial apoptotic pathway in Med-induced effects. Med is known to modulate the NFkB signaling pathway, however, our results showed that the Med-induced effects were independent of NFkB (Supplementary Figure S3c). Endoplasmic reticulum (ER) stress has a key role in the regulation of apoptosis and the ER stress-associated protein expression leads to a variety of pro-apoptotic effects.^15^ Our result also showed that Med treatment upregulated the ER stress-associated proteins in the myeloid leukemia cells K562 (Figure 3c) and U937 (Figure 3d). Our results in agreement with earlier studies indicated that the upregulation of ER stress marker proteins like Bip and p-eIF2*α* was associated with the upregulation of the pro-apoptotic CCAAT-enhancer binding protein homologous protein (CHOP) expression.^16^ Furthermore, we observed similar effects with the Med+TRAIL combination in both K562 and U937 cells in a time-dependent manner (Supplementary Figure S4a). These results collectively suggested that the Med-induced downregulation of cell survival proteins and upregulation of the pro-apoptotic and ER stress-associated protein expression could be possible mechanisms by which Med potentiates TRAIL-induced apoptosis in myeloid leukemia cells.

### Med treatment induces activation of the ROS-JNK-CHOP pathway

**Figure 4:**
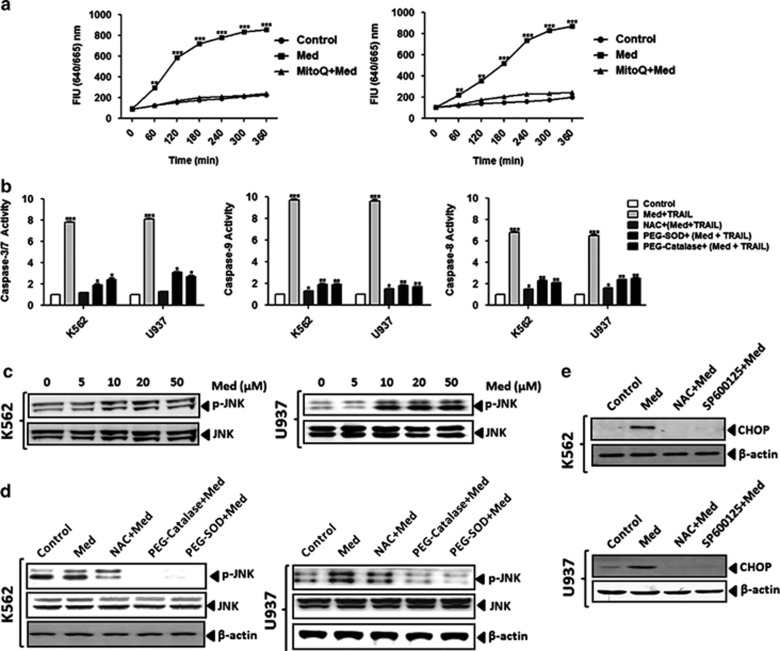
Med treatment induces activation of the ROS-JNK-CHOP pathway. (**a**) K562 or U937 cells were pretreated either with the vehicle or with the mitochondria-specific antioxidant MitoQ (50 *μ*M) for 1 h followed by the Med treatment for 6 h. (**b**) Cells were pretreated with different antioxidants to study the effect of ROS on Med-induced caspase-3/7, caspase-8, and caspase-9 activation using the Colorimetric Activity Assay kit. (**c**) Cells were treated with different doses of Med for 48 h and JNK activation was determined using western blot. (**d**) To study the ROS-dependent JNK activation, the cells were pretreated with the indicated antioxidants before Med treatment and western blot analysis of whole-cell lysates were performed. (**e**) K562 (*upper)* and U937 (*lower*) cells were pretreated with NAC and JNK inhibitor (SP600125) and western blotting was performed for CHOP expression. Representative western blots images of three independent experiments are presented. Data are presented as mean±S.D. of three independent experiments.*P>0.05, **P>0.001, ***P>0.0001 in treated groups *versus* control group

Reactive oxygen species (ROS) is known to regulate TRAIL receptor induction^17,18^ and our results showed that Med induced both DR and mitochondrial apoptotic pathways. Therefore, we investigated whether Med mediates its effects through ROS. Med induced a significant increase in the ROS, which was attenuated with pretreatment of antioxidants in both K562 and U937 cells (Supplementary Figure S4a and b). We further confirmed these findings through quantification of the mitochondrial ROS. Med induced a significant increase in the mitochondrial ROS, which was attenuated with pretreatment of the mitochondrial antioxidant MitoQ in both K562 (Figure 4a, *left*) and U937 (Figure 4a, *right*) cells, further underscoring the regulation of the mitochondrial pathway in Med-induced ROS generation. We further observed that that pretreatment of cells with the antioxidant (*N*-acetylcysteine, PEG-catalase, PEG-SOD) reduced caspase-3/7, -8, and -9 activation (Figure 4b) indicating the critical role of ROS in Med-induced effects. MAPK activation is critical to the TRAIL receptor induction.^19, 20, 21^ Therefore, we explored whether Med treatment induced activation of ERK1/2, p38 MAPK or JNK has a role in Med-induced DR5 induction. Med treatment resulted in JNK activation in a dose-dependent manner in both K562 (Figure 4c, *left*) and U937 (Figure 4c, *right*) cells, while it had no effect on the ERK1/2 or p38 expression (data not shown). Further, pretreatment with the antioxidants (*N*-acetylcysteine, PEG-catalase, PEG-SOD) completely inhibited this activation in both K562 (Figure 4d, *left*) and U937 (Figure 4d, *right*) cells, indicating that ROS generation leads to JNK activation. Studies have indicated that p53,^22^ peroxisome proliferator-activated receptor-*γ*, and CHOP mediate the upregulation of DR5.^23^ Consistent with the earlier results, we found that Med treatment induced CHOP expression in both K562 (Figure 4e, *upper*) and U937 (Figure 4e, *lower*) cells. However, pretreatment with an ROS scavenger (NAC) or the pharmacological inhibitor of JNK (SP600125) completely abolished the Med-induced CHOP expression indicating that both the ROS and JNK activation upstream is associated with CHOP induction. While inhibition of ROS, JNK, CHOP, or DR5 almost completely reversed the Med+TRAIL-induced effects (Supplementary Figure S5c) treatment of lipid raft disruptors like Nystatin or Methyl beta cyclodextrin (MBCD) had no effect (Supplementary Figure S5d), indicating no redistribution of DR5 in lipid rafts in Med-induced TRAIL sensitization. Collectively, these results suggested that Med treatment activates the ROS-JNK-CHOP signaling pathway.

### TRAIL-induced apoptosis in Med-treated cells is mediated by DR5

TRAIL-induced apoptosis is mediated through the upregulation of either death receptor 4 (DR4) or death receptor 5 (DR5).^24,25^ Therefore, we studied the respective role of these receptors in apoptosis induced by the combination of Med and TRAIL. We first examined whether Med treatment for 48 h upregulated DR4 and DR5 transcript levels in our cell lines. Med did not induce DR4 mRNA expression at significant level whereas induction of DR5 mRNA expression was observed in both the cell lines (Supplementary Figure S6a). These results were further supported by the flow-cytometric analysis of the DR4 and DR5 surface receptors (Supplementary Figure S6b). The luciferase reporter assay further confirmed a dose-dependent increase in the DR5 promoter activity (Supplementary Figure S6c). Further, these findings were confirmed with a dose- and time-dependent increase in the DR5 protein levels upon Med treatment (Supplementary Figure S6d).

**Figure 5:**
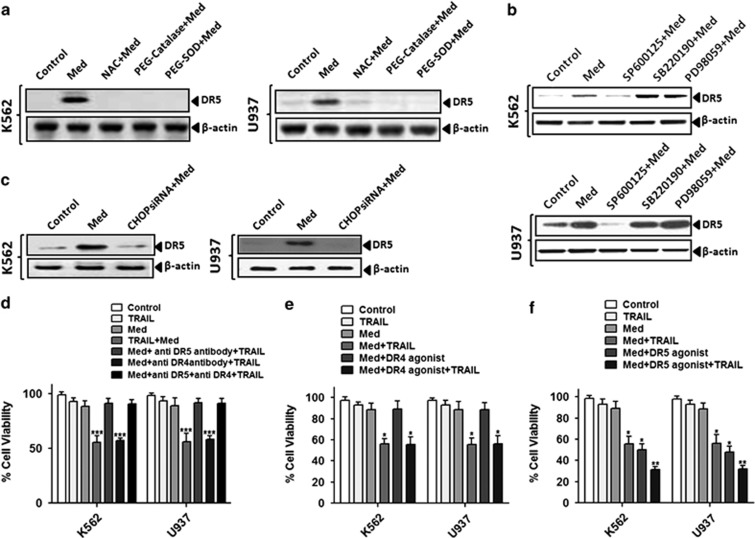
TRAIL-induced apoptosis in Med-treated cells is mediated through DR5. (**a**) K562 and U937 cells were pretreated with indicated antioxidants for 1 h before Med treatment and cell lysate was prepared for western blot analysis. (**b**) K562 and U937 cells were pretreated with inhibitors of JNK (SP600125), p38 MAPK (SB202190), and ERK1/2 (PD98059) before Med treatment for 1 h and cell lysates were prepared for performing western blotting. (**c**) K562 (*left*) and U937 (*right*) cells were transfected with CHOP siRNA and then treated with Med and cell lysates were prepared for performing western blotting for DR5 antibody. (**d**) K562 and U937 cells were treated with TRAIL or Med or combination of Med and TRAIL or Med and TRAIL with DR5 antagonist or Med and TRAIL with DR4 antagonist or Med and TRAIL with DR4 and DR5 antagonist together and cell viability was measured as described under ‘Material and Methods'. (**e**) K562 and U937 cells were treated with Med or TRAIL or combination of Med and TRAIL or Med with DR4 agonist or Med and TRAIL with DR4 agonist and cell viability was measured as described under ‘Material and Methods. (**f**) K562 and U937 cells were treated with Med or TRAIL or Med with DR5 agonist or Med and TRAIL with DR5 agonist and cell viability was measured as described under ‘Material and Methods. Data are presented as mean±S.D. *P>0.05, **P>0.001, ***P>0.0001 in treated groups *versus* control group

As the activation of the ROS-JNK-CHOP pathways was observed upon Med treatment, we further explored whether it was directly linked to the DR5 activation. Our results showed that the pretreatment of the ROS scavengers completely abolished DR5 expression, suggesting that ROS has a critical role in mediating the effects of Med on TRAIL-induced apoptosis in both K562 (Figure 5a, *left*) and U937 cells (Figure 5a, *right*). Next, to investigate the direct involvement of JNK, ERK1/2, or p38 MAPK in the Med-induced DR5 induction, we used the pharmacological inhibitors of JNK (SP600125), p38 MAPK (SB202190), and ERK1/2 (PD98059) and checked Med-induced DR5 induction. We observed that pretreatment of the JNK-specific inhibitor (SP600125) completely suppressed the Med-induced DR5 induction in both K562 (Figure 5b, *upper*) and U937 (Figure 5b, *lower*) cells; while the p38 MAPK (SB220190) and ERK (PD98059) inhibitors had no effect suggesting the critical role of that JNK for DR5 induction. Similarly, genomic inhibition of CHOP using specific CHOP siRNA (Supplementary Figure S5b) significantly suppressed Med-induced DR5 upregulation in both K562 (Figure 5c, *left*) and U937 (Figure 5c, *right*) cells. These results further confirmed the Med-induced activation of the ROS-JNK-CHOP-DR5 signaling cascade.

Several agonist antibodies targeting either DR4 or DR5 are currently in clinical development.^26^ Therefore, we next assessed the impact of antagonistic antibodies directed against DR4 or DR5 on the viability of myeloid leukemia cells treated with the Med+TRAIL combination. In both the tested cell lines, DR4 inhibition only partially reversed the reduction in cell viability induced by the combination of Med and TRAIL (Figure 5d). However, DR5 inhibition almost completely abrogated the effect of TRAIL (Figure 5d). Inhibition of both DR4 and DR5 did not further enhance the effect observed with inhibition of DR5 alone (Figure 5d). To further confirm the involvement of DR5 in this process, we evaluated the effect of a DR5 agonistic antibody on cell viability. Mimicking results obtained with TRAIL, the DR4 agonist had no or little effect on K562 and U937 cells viability (Figure 5e). More importantly, the DR5 agonist significantly reduced cell viability in Med-treated K562 and U937 cells (Figure 5f). Furthermore, the genomic inhibition of DR5 further confirmed these results (Supplementary Figure S7a and b). The Med-induced DR5 activation appeared to be specific for myeloid leukemia cells (Supplementary Figure S7c). Collectively, these data underscore the critical role of DR5 and the sequential activation of the ROS-JNK-CHOP-DR5 signaling cascade in Med potentiation of TRAIL-induced cell death.

### Concomitant treatment with Med and TRAIL induces cell death in primary AML and BC-CML cells but has no toxic effects in primary human PBMCs

To confirm the results *in vitro*, we treated primary AML (*N*=8) and blast crisis chronic myeloid leukemia (BC-CML, *N*=7) primary cells with the Med and TRAIL combination for 48 h. The combination induced robust cell death in all the samples tested (Supplementary Figure S7d and e).

TRAIL is considered to have no toxic effects in normal cells. However, it is necessary to assess the effects when TRAIL is used in combination with other treatments because the same molecular mechanisms may be involved in TRAIL resistance of both normal and tumor cells, resulting in sensitization of otherwise resistant normal cells.

**Figure 6:**
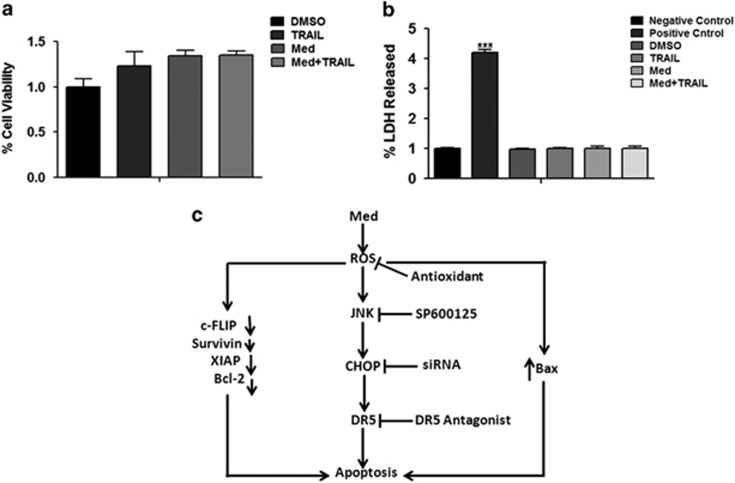
Treatment with Med and TRAIL is not toxic for human PBMCs. Primary human PBMCs (1 × 10^6^cells/well) were incubated in a culture medium containing DMSO or Med (20 *μ*M) or Med (20 *μ*M)+TRAIL (2.5 ng/ml) (*n*=12 per group) for 48 h. Cell viability was then assessed using a CCK-8 assay (**a**) and cell death was assessed by an LDH release assay (**b**). Data are presented as mean±S.D. *P>0.05, **P>0.001, ***P>0.0001 in treated groups *versus* control group. (**c**) A schematic model describing the mechanism by which Med potentiates TRAIL-induced apoptosis in myeloid leukemia cells

To address this issue, primary normal human PBMCs were pretreated with DMSO or 20 *μ*M of Med along with 2.5 ng/ml of human recombinant TRAIL for 48 h. The cell viability and cell death (LDH release) assays indicated no significant difference neither in cell viabilities nor in LDH release in cells treated with Med and TRAIL, either alone or in combination, compared with that of the control (DMSO) group (Figures 6a and b). These results confirmed that combination of Med and TRAIL is not toxic for primary normal human PBMCs.

## Discussion

The selective induction of apoptosis by TRAIL and TRAIL receptor agonists have led to the clinical development of these agents as promising anticancer agents. However, in myeloid leukemia, development of TRAIL resistance is a major bottleneck limiting therapeutic efficacy. The best clinical response obtained with the recombinant human TRAIL therapy is stable disease. Therefore, the best clinical use of TRAIL seems to be in combination with TRAIL sensitizing agents. In this study, we investigated the ability of Med, a phytoalexin, to modulate TRAIL signaling in cancer cells. Our findings indicate that Med potentiates TRAIL-induced apoptosis in myeloid leukemia cells by downregulating cell survival proteins, upregulating cell death proteins, and inducing DR5 expression through the activation of the ROS-JNK-CHOP pathway.

Med-induced TRAIL sensitization involved both the extrinsic and the intrinsic pathways of apoptosis, as evidenced by a robust induction of cleaved caspase-8 and caspase-9. This finding suggests that myeloid leukemia cells are type II cells capable of amplifying apoptotic signaling initiated by DR's through the recruitment of the mitochondrial pathway, similar to other cell types.^26^

Overexpression of Bcl-2 or the Bcl-2 family of antiapoptotic proteins is known to induce TRAIL resistance in cancer cells.^27^ In our study, Med treatment downregulated Bcl-2 and BclxL was suppressed by Med treatment. Med treatment also suppressed the potent cellular caspase inhibitor XIAP, thereby potentiating TRAIL-induced apoptotic cell death in agreement with earlier studies.^28,29^ In addition, Med treatment also decreased the expression of other cell survival proteins like survivin and c-FLIP linked to TRAIL resistance.^30^ Med treatment induced G2/M cell-cycle arrest and increased the tBid and Bax expression along with release of cytosolic cytochrome C and Smac/Diablo confirming activation of both the DR and mitochondrial apoptotic pathways and appeared to aid in TRAIL sensitization.^31^ Furthermore, Med treatment upregulated pro-apoptotic ER stress-associated proteins like Bip, p-eIF2*α*, and CHOP expression known to have critical role in TRAIL sensitization.^17,18^ Our results are in agreement with previous studies showing that regulation of the intracellular pro- and antiapoptotic protein ratio is essential for TRAIL sensitization.^27, 28, 29, 30, 31, 32^

ROS has a major role as a mediator of apoptosis^33,34^ and is known to be involved in TRAIL sensitization through the upregulation of DR5 by cancer chemopreventive agents.^17,18^ In the current study, Med induced generation of mitochondrial ROS and ROS mediated upregulation of DR5 in agreement with previous studies.^33, 34, 35, 36^ We found that quenching ROS by antioxidants abolished Med-induced potentiation of TRAIL-induced apoptosis indicating the critical role of ROS in modulation of TRAIL receptor DR5. Activation of stress-activated proteins such as JNK is known to enhance TRAIL-induced apoptosis.^20,21^ Our findings provide evidence that activation of JNK by Med upregulates DR5, which may further lead to an increase in TRAIL-induced apoptosis. Med was found to be ineffective in activating ERK1/2 and GSK-3*β*, although there are reports suggesting that ROS can lead to induction of ERK1/2.^37^ In our study, Med induced TRAIL receptors independently of ERK1/2. JNK activation is also found to be involved in the induction of DR5 expression and we found that JNK was activated by Med, and pretreatment with SP600125, a specific inhibitor of JNK, could reduce the Med-induced DR5 upregulation, suggesting that novel mechanisms may be responsible for Med-induced DR5 upregulation. Our results clearly show that Med induces ROS, which leads to the upregulation of CHOP through the activation of JNK (Figure 6c).

In this study, DR5 upregulation was also mediated through CHOP induction. We found that Med-induced CHOP and that the gene silencing of CHOP by siRNA blocked the effect of Med on the induction of DRs and on TRAIL- induced apoptosis. Our findings are similar to those of other studies that indicated that CHOP binds to the DR5 promoter and upregulates this receptor expression.^38^ Inhibition of the NFkB signaling pathway and p53 induction have been linked to DR5 upregulation.^39, 40, 41^ However, our results showed that the Med-induced upregulation of TRAIL receptor DR5 was independent of p53 and NFkB as it induced increased DR5 expression in p53-deficient K562 cells in agreement with earlier studies.^42,43^ Consistent with this finding, an agonist antibody specific for DR5 also reduced cell viability in cells pretreated with Med. These data are in agreement with the earlier studies of a greater contribution of DR5 than DR4 to apoptosis induction in cancer cells expressing both receptors. As both DR4 and DR5 agonists are under clinical development our results underscore the importance of DR5 in myeloid leukemia cells. Moreover, TRAIL-induced reduction in cell viability was strongly attenuated when cells were incubated with an antagonist antibody against DR5, but not DR4. Finally, the TRAIL-induced cytotoxicity is an important concern regarding its use of TRAIL and DR agonists. It is therefore crucial to ensure that a treatment used to sensitize tumor cells to TRAIL-induced apoptosis does not induce toxicity in primary normal human PBMCs. As mice only harbors one apoptosis-inducing TRAIL receptor, equally homologous to both human apoptosis-inducing TRAIL receptors, DR4 and DR5,^44^ classical murine models are not suited to evaluate the toxicity of TRAIL-based treatments. Therefore, we evaluated the effect of TRAIL or Med alone or in combination *in vitro* in primary AML, BC-CML cells. The Med+TRAIL combination induced significant apoptosis in AML and BC-CML primary cells; but did not affect cell viability or induce significant cytotoxicity in primary normal human PBMCs, underscoring the translational relevance of the combination.

## Conclusion

Our results show for the first time that the phytoalexin Med sensitizes human myeloid leukemia cell lines to TRAIL-induced cell death by activation of both the intrinsic and the extrinsic pathways of apoptosis, while this combination is not toxic for primary human PBMCs. Apoptosis induction in cells treated with this combination is mediated by the DR5, but not by the DR4 receptor. Furthermore, we show that inhibition of antiapoptotic proteins by Med has an important role in this sensitization process. As several natural agent TRAIL agonists are currently under clinical development, these results in human myeloid leukemia cell lines and primary PBMCs provide a rationale for testing the combination of Med and TRAIL agonists in management of myeloid leukemia.

## Materials and Methods

### Reagents

Medicarpin (Med), a naturally occurring phytoalexin was synthesized in gram scale at the medicinal process chemistry division of the CSIR-Central Drug Research Institute, India as per a standardized procedure.^45^ The Med stock (20 mM in DMSO, stored at −20°C) solution was diluted in cell culture media for experimental use. The Super killer TRAIL/Apo2L and the antagonistic antibodies against DR4 (HS101) and DR5 (HS201) were purchased from Alexis Biosciences (San Diego, CA, USA). The primary antibodies against DR4, c-FLIP, Bid, tBid, Cytochrome C, Smac/Diablo, and *β*-actin were purchased from Santa Cruz Biotechnology, Inc. (Santa Cruz, CA, USA), while the antibodies against DR5, Survivin, CHOP, XIAP, JNK, p-JNK, Bip, eIF2*α*, p-eIF2*α*, cleaved caspase-8, cleaved caspase-9, cleaved caspase-3, and cleaved caspase-7 were purchased from Cell Signaling Technology (Boston, MA, USA). Primary antibodies against Bcl-2 and Bax and the 7AAD/Annexin-based cell death assay kit were purchased from BD Biosciences (San Jose, CA, USA). All the other biochemicals were from Sigma (St Louis, MO, USA) unless otherwise stated.

### Cell culture and transfection

All the cell lines were obtained from the American Type Culture Collection (ATCC; Manassas, VA, USA). The cell lines K562, LAMA-84 (chronic myeloid leukemia cell lines), U937, OCIAML-3 (the AML cell lines) maintained in RPMI-1640 medium supplemented with 10% fetal bovine serum both from Gibco (Carlsbad, CA, USA) along with 1% penicillin and streptomycin from Sigma in a humidified incubator at 37°C with 5% CO_2_. The peripheral blood samples were obtained from normal healthy donors and CD34-positive AML or BC-AML at the King George Medical University, Lucknow, India, after written informed consent in compliance with the Declaration of Helsinki 2002. PBMCs from all the donors were separated by ficoll-hypaque density gradient (1.0 g/ml) centrifugation method. Subsequently, the isolated cells (10^6^/ml) were cultured in complete RPMI-1640 medium supplemented with 10% FBS. Blasts were verified by immunofluorescence flow cytometry to be composed >80% CD34-positive cells. Primary blast cells (10^6^/ml) were cultured in the Iscove's modified Dulbecco's medium (IMDM) containing 20% FCS, 1 mM L-glutamine, and streptomycin/penicillin. All the cell lines or the primary cells were treated with either DMSO or 20 *μ*M Med for the indicated time points or a combination of 20 *μ*M Med with or without 2.5 ng/ml TRAIL (*n*=12 per group) for 48 h. Transfections were carried out in the cell lines using the Lipofectamine 2000 (Invitrogen, Carlsbad, CA, USA).

### Determination of cell viability and LDH Release

The cell viability was quantified using the Cell Counting Kit-8 according to the manufacturer's instructions (Dojindo, Kumamoto, Japan). The LDH release was measured using the Cytotoxicity Detection KitPlus (Roche, Mannheim, Germany) following the manufacturer's instructions.

### Cell cycle and cell death analysis

For the cellc-ycle analysis, cells were harvested after treatment and cells were harvested and washed twice with PBS and fixed in 70% ethanol at −20°C overnight. Fixed cells were washed with 1X PBS and resuspended in propidium iodide solution containing PI (50 *μ*g/ml) and RNase A (50 *μ*g/ml) diluted in PBS and incubated for 30 min at RT. Stained cells were sorted for specific cell-cycle phase arrest in FACS Caliber and data were analyzed using the Modfit LT 3.0 software (Verity Software House, Topsham, ME, USA). Cell death was measured by using the 7AAD/Annexin V-based flow-cytometric kit (BD Bioscience) and the Apoptosis detection kit (Invitrogen) according to the manufacturer's instructions. The samples were analyzed for live, necrotic, early, and late apoptotic cells using FACS Caliber (BD Bioscience) flow cytometer. Preliminary Caspase activity was assessed using the Caspase-Glo-3/7, -8, and -9 assay kits (Promega, Leiden, The Netherlands) according to the manufacturer's instructions.

### Measurement of ROS

The ROS and the mitochondrial ROS were measured using a previously standardized protocol.^46^ Briefly, 1 × 10^6^ cells were incubated either with 5 *μ*M of cell-permeant, fluorogenic ROS sensor CellROX Deep Red reagent or with 10 *μ*M of MitoPY1 (Invitrogen) in the culture media for 30 min at 37°C and subsequently the indicated treatments were initiated. Fluorescence was measured either at an excitation wavelength of 640 nm and an emission wavelength of 665 nm or at an excitation wavelength of 514 nm and an emission wavelength of 530 nm using the Fluostar Omega spectrofluorometer (BMG Technologies, Offenburg, Germany).

### Real-time PCR

The mRNA extraction and the quantitative PCR were carried out according to a standardized protocol.^46^ Briefly, mRNA from the samples was extracted using Trizol (Invitrogen) as per the manufacturer's instructions. The isolated mRNA was converted to cDNA using a cDNA synthesis kit (Fermentas, Austin, TX, USA). For evaluation of the level of gene expression, real-time PCR with SYBRGreen dye was used in LC480 II light cycler real-time PCR machine (Roche Molecular Biochemicals, Indianapolis, IA, USA). The real-time PCR mixture contained 10 *μ*l Syber Green Super Mix, 100 nM of each primer (DR4: forward 5′-TGTCAGTGCAAACCAGGAAC-3′ and reverse 5′-TGCTCAGAGACGAAAGTGGA-3′) and DR5: forward 5′-TGCAGCCGTAGTCTTGATTG-3′ and reverse 5′-GCACCAAGTCTGCAAAGTCA-3′) and 1 *μ*l cDNA. All samples were run in triplicates and each experiment was repeated at least three times independently. Each sample was normalized on the basis of GAPDH.

### Cloning of the DR5 promoter and luciferase assay

The DR5 promoter cloning was done following a previously standardized protocol.^47, 48, 49^ The pDR5/mtCHOP was generated using a site-directed mutagenesis kit (Stratagene, La Jolla, CA, USA). Cells were harvested 48 h after transfection and assayed using the Dual Luciferase Reporter Assay System (Promega, Madison, WI, USA).

### Analysis of cell surface expression of DR4 and DR5

Control and Med-treated cells were stained with phycoerythrin-conjugated mouse monoclonal anti-human DR5 or DR4 (R&D Systems, Minneapolis, MN, USA) for 45 min at 4°C according to the manufacturer's instructions and analyzed by flow cytometry. The phycoerythrin-conjugated mouse IgG2B was used as an isotype control.

### Western blotting

Cell lysates were prepared in radioimmunoprecipitation assay (RIPA) lysis buffer (50 mmol/l Tris-HCl, 150 mmol/l NaCl, 1% NP-40, 0.5% SDS, and 1% deoxycholic acid) and the western blotting analysis was conducted as per a standardized procedure.^50^ Thedilutions of the primary and secondary antibodies are 1 : 2000 and 1 : 5000, respectively. Blots were developed using chemiluminescent substrate (Millipore, Billerica, CA, USA).

### Statistical analysis

All the values are represented as mean±S.E.M. from at least three independent experiments. Data were analyzed using one-way ANOVA followed by Newman Keuls comparison test. Values with *P<0.05 were considered to be significant.

## Abbreviations

Med: Medicarpin
TRAIL: tumor necrosis factor-related apoptosis-inducing ligand
LDH: lactate dehydrogenase
tBid: truncated Bid
AML: acute myeloid leukemia
CML: chronic myeloid leukemia
BC-CML: blast crisis chronic myeloid leukemia
DR: death receptor
DR4: death receptor 4
DR5: death receptor 5
DMSO: dimethyl sulfoxide
JNK: c-Jun N-terminal kinases
ROS: reactive oxygen species
CHOP: CCAAT-enhancer binding protein homologous protein
MBCD: Methyl Beta Cyclo Dextrin
