# Supplementary material for: Medicarpin, a legume phytoalexin sensitizes myeloid leukemia cells to TRAIL-induced apoptosis through the induction of DR5 and activation of the ROS-JNK-CHOP pathway
Source: Cell Death Dis. 2014 Oct 16;5(10):e1465–. doi: 10.1038/cddis.2014.429 (PMC4237253; doi:10.1038/cddis.2014.429)
Supplement: Supplementary Figures [file cddis2014429x1.pdf]

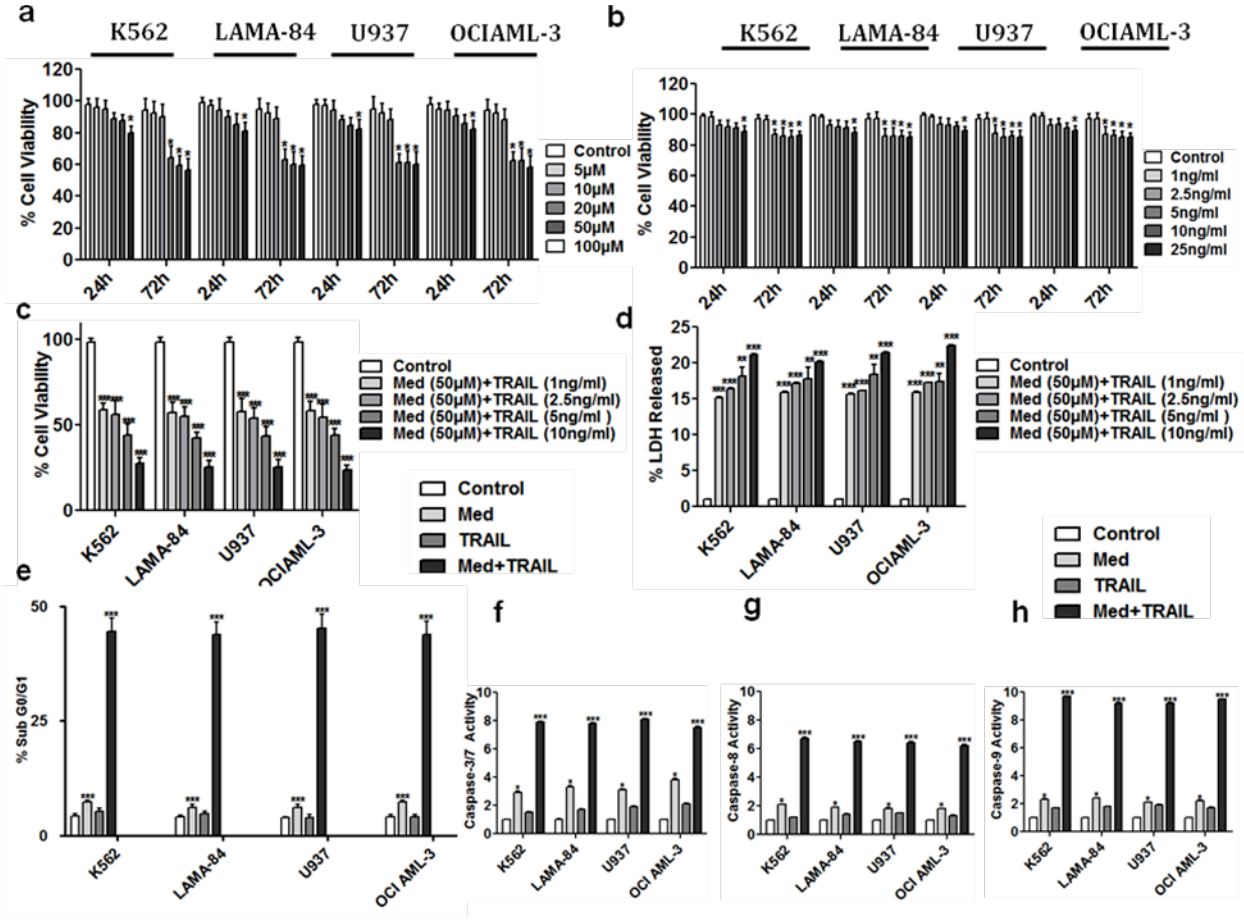

Supplementary Figure S1. Med induces TRAIL sensitization in myeloid leukemia cells through the extrinsic and intrinscic apoptotic pathway. Cytotoxic effect of Med and TRAIL on myeloid leukemia cells. K562, LAMA-84, U937 and OCIAML-3 were treated with different doses of Med **(a)** and TRAIL **(b)** for 24h and 72h and Cell viability was measured using CCK-8. K562, LAMA-84, U937 and OCIAML-3 cells were treated with 50μM Med and indicated doses of TRAIL for 48h and cell viability **(c)** and LDH release **(d)** assay was performed. **(e)** K562, LAMA-84, U937 and OCIAML-3 cells were treated with 20μM Med and 2.5ng/ml TRAIL for 48h. Cells were stained with PI and then analyzed by FACS. K562, LAMA-84, U937 and OCIAML-3 cells were treated with 20μM Med and 2.5ng/ml TRAIL for 48h caspase-3 **(f)** caspase-8 **(g)** and caspase-9 **(h)** activity was measured using the Colorimetric Activity Assay kits. Data of three independent experiments are presented as mean ± S.D. *p>0.05, **p>0.001, ***p>0.0001 in treated groups versus control group.

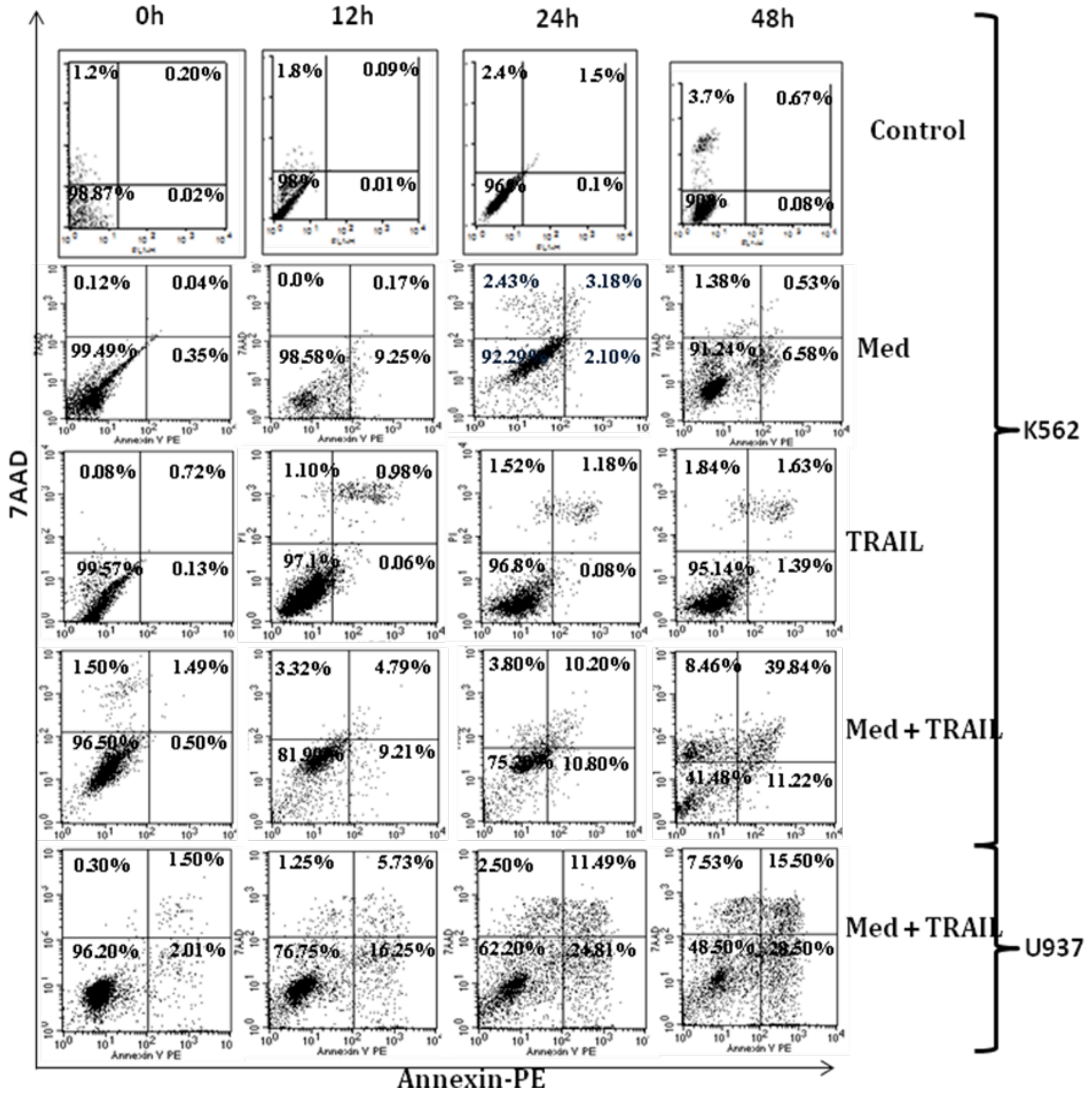

Supplementary Figure S2. The Med+TRAIL combination induces apoptotic cell death in myeloid leukemia cells. K562 and U937 cells were treated with Med, TRAIL and combination of Med and TRAIL for the indicated time points. Percentage of apoptotic cell was measured by 7AAD / Annexin-V staining. Representataive images of three independent experiments are presented.

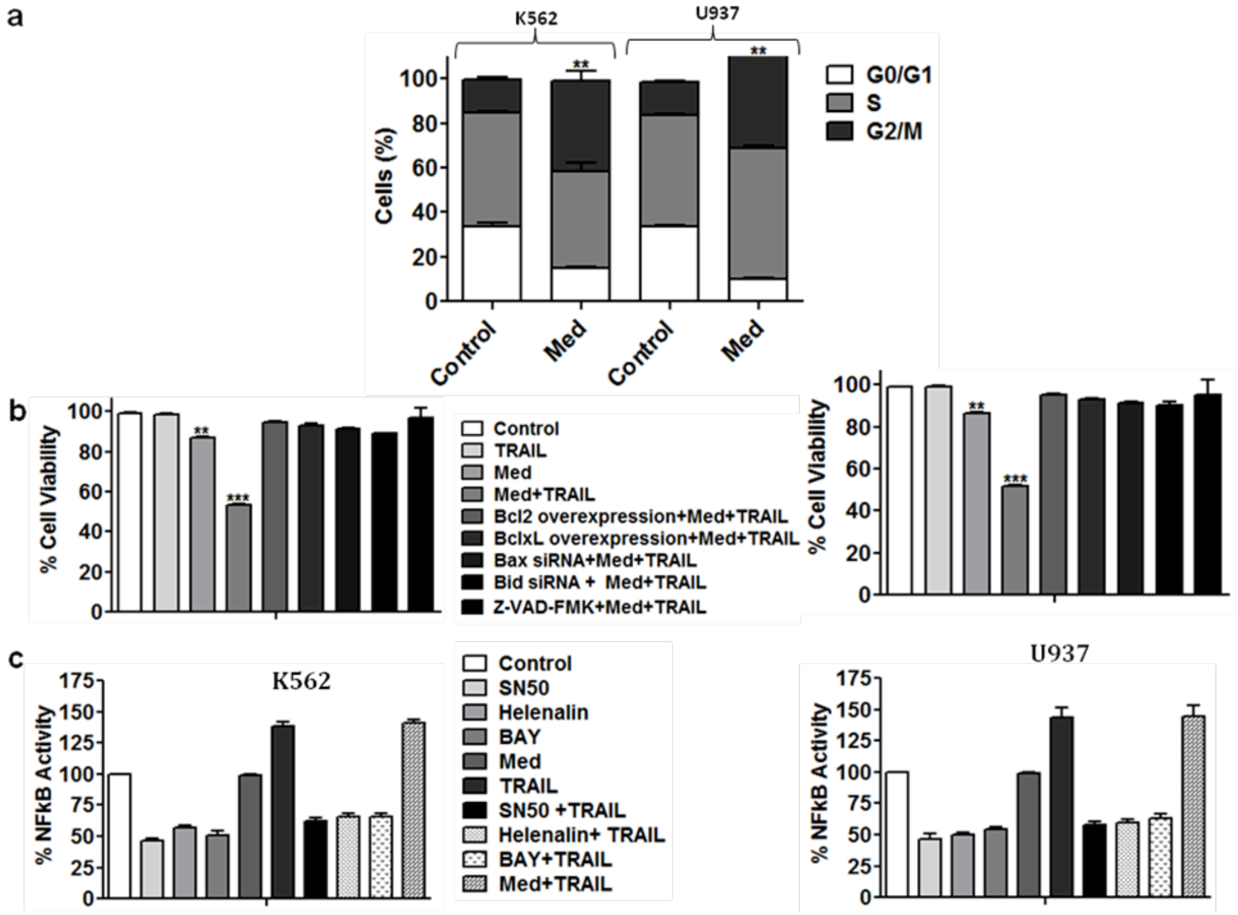

Supplementary Figure S3. Med treatment induces G2/M cell cycle arrest through modulation of Bcl-2 family proteins without influencing the NFkB pathway. To study the effect of Med on cell cycle, K562 and U937 cells were treated with Med and cell cycle analysis was performed by PI staining **(a)**. K562 and U937 cells **(b)** were transfected overexpression and knockdown of Bcl-2 expression and then treated with combination of Med and TRAIL to study the effect of modulation of Bcl2 family proteins on Med induced TRAIL sensitization. **(c)** To study the effect of Med on NFkB activity, K562 and U937 cells were Cells were transfected with either the pTATA-luc or the pNF-kB-luc plasmids. After a recovery period of 36 hours, transfected cells were treated with various concentrations of Medicarpin (20µM), SN50 (50µM), Helenalin (5µM) or BAY 11-7082 for 2 hours followed by a further 10 hours in the presence or absence of TRAIL (2.5ng/mL). Luciferase assays were performed using the Dual Luciferase Assay System (Promega). Data of three independent experiments are are presented as mean ± S.D. *p>0.05, **p>0.001, ***p>0.0001 in treated groups versus control group.

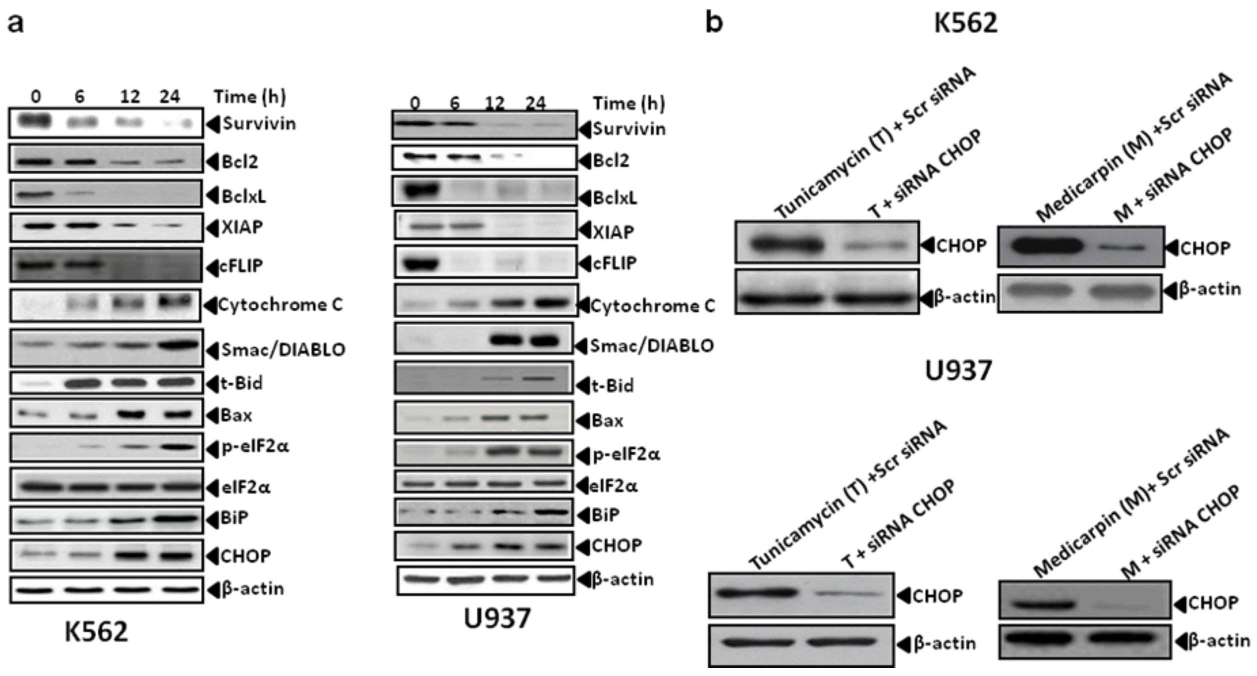

Supplementary Figure S4. The Med+TRAIL combination induces similar changes in the protein expression as Med (a) K562 and U937 cells were treated with combination for Med and TRAIL for indicated time points. After different treatment hours, cell lysates were prepared for western blot analysis. **(b)** K562 and U937 cells were transfected with Scrambled and CHOP siRNA and after transfection cells were treated with tunicamycin and Med and then cell lysates were prepared and analysed for CHOP expression by western blot analysis. Representative images of three independent experiments are presented.

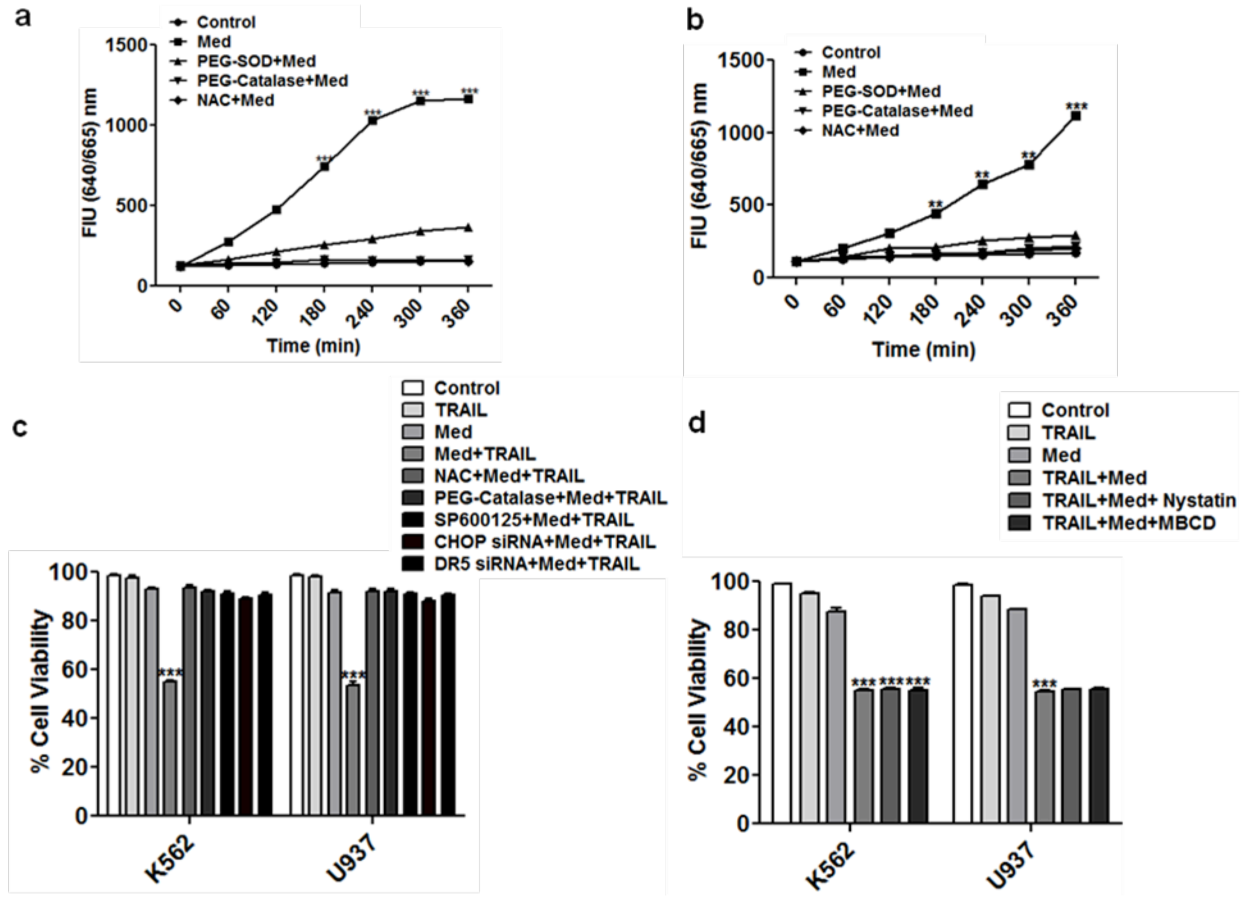

Supplementary Figure S5. Med induces ROS generation and activates the ROS-JNK-CHOP-DR5 pathway. For ROS measurement, K562 **(a)** and U937 **(b)** cells were pretreated with indicated antioxidants for 1h and then treated with Med to study the ROS generation at different time points. ROS was measured using the cell-permeant, fluorogenic ROS sensor CellROX™ Deep Red reagent. To study the effect of ROS, JNK, CHOP and DR5 on Med induced sensitization of TRAIL induced Cell death K562 and U937 cells **(c)** were incubated with ROS inhibitor Catalase, JNK inhibitor SP600125, and transfected with CHOP siRNA and DR5siRNA and then further treated with combination of TRAIL and Med and then cell viability was measured using MTT assay kit. To study the effect of Lipid raft disruptors on Med induced TRAIL Sensitization. K562 and U937 cells **(d)** were treated with either Med or TRAIL alone for 48h. Cells were treated with (20 ng/mL) nystatin or (5 mMol/ L) methyl beta cyclo dextrin for the last 12 h and the % cell viability was assessed by 7AAD / Annexin staining. Data of three independent experiments are presented as mean ± S.D. *p>0.05, **p>0.001, ***p>0.0001 in treated groups versus control group.

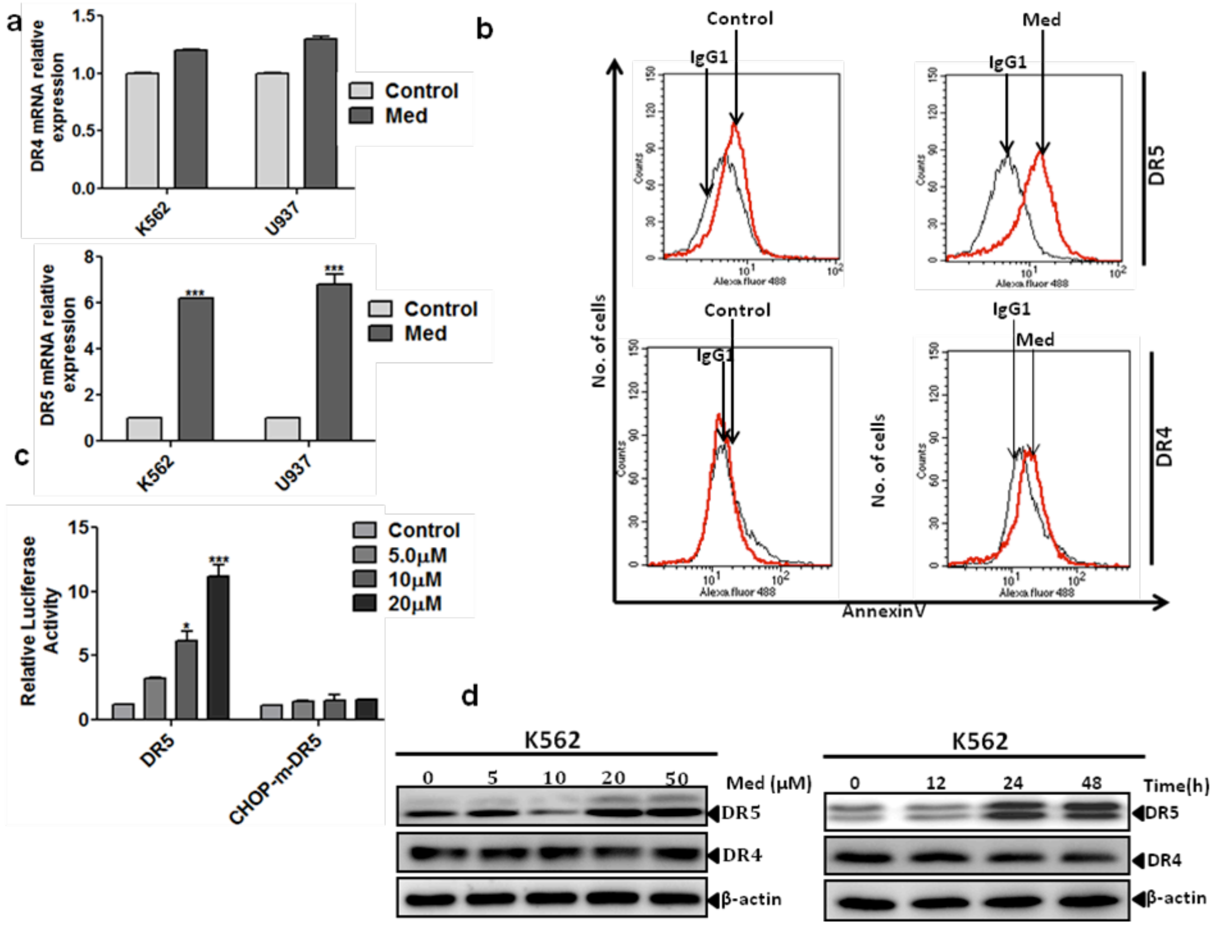

Supplementary Figure S6. DR5 activation is critical to Med induced effects. Effect of Med on DR5 promoter activity and DR5 protein and mRNA expression. **(a)** K562 and U937 cells were treated with 20μM Med for 48h and fold increase in DR4 and DR5 was measured. **(b)** K562 cells were treated with 20μM Med for 48h and DR5 and DR4 surface expression was analyzed by FACS analysis. **(c)** K562 cells were transfected with DR5 or CHOP-mutated-DR5 and then treated with the indicated concentrations of Medicarpin. After 48h, cell lysates were assayed for luciferase activity. **(d)** K562 cells were treated with indicated doses (*left*) and indicated time points (*right*) and cell lysates were prepared for western blot analysis. Data of three independent experiments are presented as mean ± S.D. *p>0.05, **p>0.001, ***p>0.0001 in treated groups versus control group.

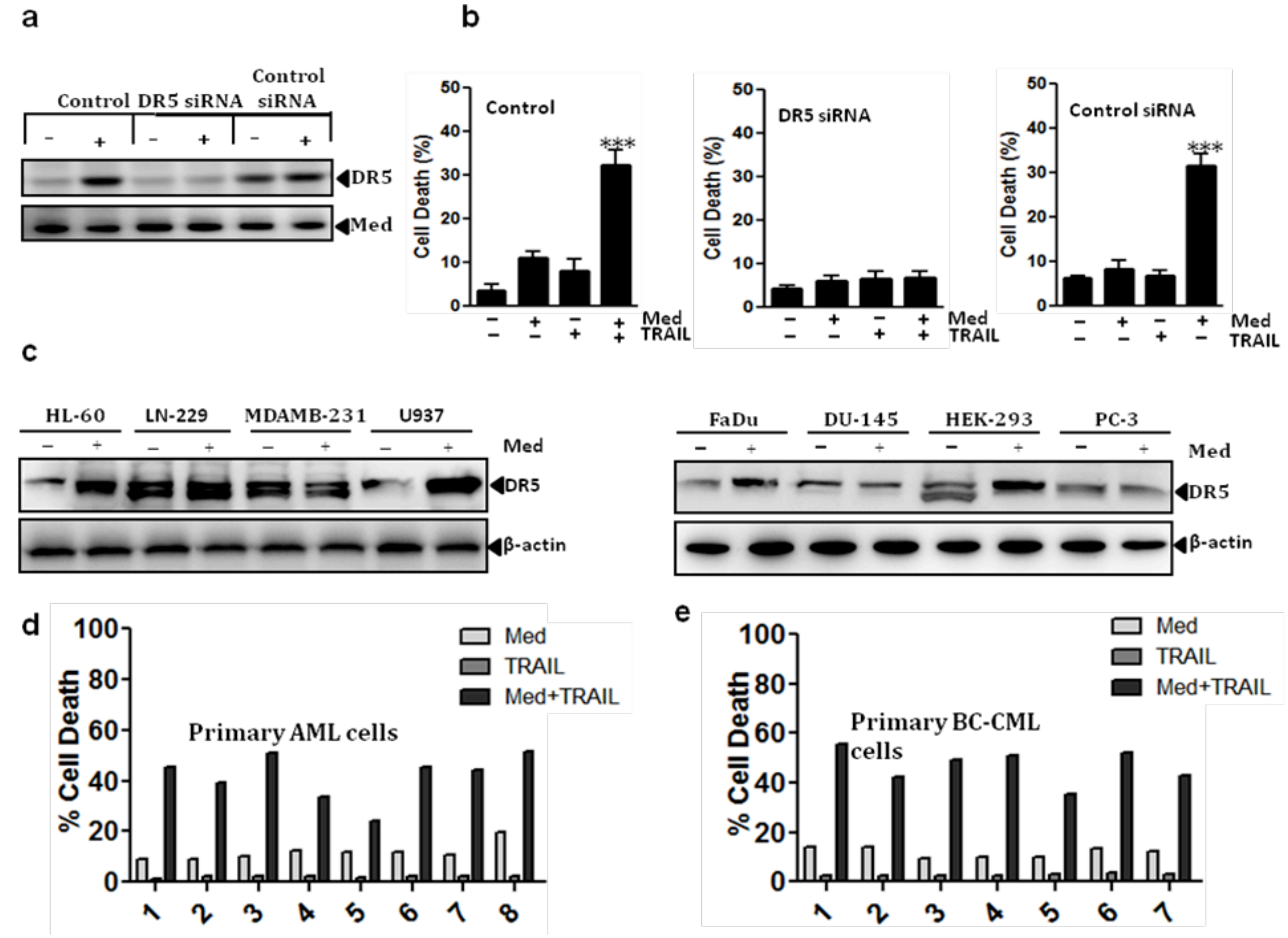

Supplementary Figure S7. Med induced TRAIL sensitization is specific for myeloid leukemia cell lines and primary cells. Med induces DR5 mediated apoptosis. **(a)** K562 cells were transfected with DR5 siRNA or Control siRNA and then non-transfected and transfected cells were treated with 20μM Med and western blot analysis was performed after 48h of treatment. **(b)** Transfected K562 cells were treated with 20μM Med for 48h and Annexin-V/PI staining was performed to measure % of apoptotic cells. **(c)** HL60, U937, DU145, PC3, MDAMB-231, LN229, FaDu and HEK-293 cells were treated with 20μM of Med for 48h and western blot analysis was performed using antibody against DR5. To further study the effect of combination of Med and TRAIL on AML and CML patient derived PBMCs. The peripheral blood samples derived from CD34 positive acute myeloid leukemia (AML) **(d)** or blast crisis – chronic myeloid leukemia (BC-AML) **(e)** were treated with Med or TRAIL or combination of Med and TRAIL and percentage of apoptotic cell were measured by

Annexin-V/PI staining. Data of three independent experiments are presented as mean ± S.D. *$p>0.05$, **$p>0.001$, ***$p>0.0001$ in treated groups versus control group.
